# Challenges of Revisional Metabolic and Bariatric Surgery: A Comprehensive Guide to Unraveling the Complexities and Solutions of Revisional Bariatric Procedures

**DOI:** 10.3390/jcm13113104

**Published:** 2024-05-25

**Authors:** Lorna A. Evans, Rocio Castillo-Larios, Jorge Cornejo, Enrique F. Elli

**Affiliations:** Department of Surgery, Mayo Clinic Florida, 4500 San Pablo Rd., Jacksonville, FL 32224, USA

**Keywords:** revisional surgery, metabolic and bariatric surgery, minimally invasive surgery, robotic surgery, adjustable gastric banding, sleeve gastrectomy, Roux-en-Y gastric bypass, duodenal switch, single-anastomosis duodeno–ileal bypass

## Abstract

Revisional metabolic and bariatric surgery (RMBS) presents unique challenges in addressing weight loss failure or complications arising from initial bariatric procedures. This review aims to explore the complexities and solutions associated with revisional bariatric procedures comprehensively, offering insights into the evolving terrain of metabolic and bariatric surgery. A literature review is conducted to identify pertinent studies and expert opinions regarding RMBS. Methodological approaches, patient selection criteria, surgical techniques, preoperative assessments, and postoperative management strategies are synthesized to provide a comprehensive overview of current practices and advancements in the field, including institutional protocols. This review synthesizes key findings regarding the challenges encountered in RMBS, including the underlying causes of primary procedure failure, anatomical complexities, technical considerations, and assessments of surgical outcomes. Additionally, patient outcomes, complication rates, and long-term success are presented, along with institutional approaches to patient assessment and procedure selection. This review provides valuable insights for clinicians grappling with the complexities of RMBS. A comprehensive understanding of patient selection, surgical techniques, preoperative management, and postoperative care is crucial for enhancing outcomes and ensuring patient satisfaction in the field of metabolic bariatric surgery.

## 1. Introduction

Obesity has become a worldwide epidemic in the last few years. According to the World Health Organization, 16% of the worldwide adult population was obese in 2022 [1]. In the last 20 years (2000–2020), obesity rates in the US went from 30.5% to 42.4%, and the prevalence of severe obesity (BMI > 35 kg/m^2^) increased from 4.7% to 9.2% [2]. 

By 2030, obesity rates in the US are predicted to reach 51.1% [3]. Obesity is a risk factor for other diseases, such as heart disease, stroke, type 2 diabetes, and some types of cancer, which are among the world’s leading causes of death [4,5,6,7]. Even though obesity may not directly cause death, it has been recognized as one of the most important risk factors for mortality, with 4.72 million deaths attributable to it [8]. Worldwide, each year, 2.8 million people die as a result of being overweight or obese [9]. 

Metabolic and bariatric surgery (MBS) has been demonstrated to be the most effective long-term treatment for obesity, leading to both significant weight loss (WL) and comorbidity resolution [10,11]. As obesity rates increase, it is not surprising that bariatric procedures have also significantly increased during the last few years [10]. Between 2006 and 2009, bariatric procedures went from 4.35/100,000 to 70.6/10,000 [12]. 

Although MBS rates were steady between 2010 and 2015 [12], just in 2019, around 256.000 MBS were performed in the US [13]. Worldwide, it is estimated that nearly 580,000 people undergo MBS annually [14]. Despite all the beneficial effects of MBS, these procedures are not free from complications, and around 28% of all MBS will require revision [15]. Revisional metabolic and bariatric surgeries comprised 16.8% of all bariatric procedures in 2019 [16]. Moreover, since 2011, there has been a 311% rise in RMBS [17]. 

Due to the presence of adhesions and anatomical distortion, revisional metabolic and bariatric surgery (RMBS) is more complex than primary MBS and has been associated with higher overall morbidity. Longer operative times and length of hospital stay, greater blood loss and risk for Intensive Care Unit stay, and more postoperative complications, readmissions, and reoperations have been reported for revisional procedures [18]. However, RMBS can be safely performed with acceptable outcomes in most cases [19], when performed by experienced bariatric surgeons in bariatric centers that have the resources to manage possible complications [20].

## 2. Indications for RMBS

There is no official definition for “failure of MBS.” However, most surgeons describe the failure of MBS in terms of WL [21]. Usually, a successful MBS is defined as a WL > 50% of excess body weight [21,22]. Therefore, insufficient weight loss (IWL), defined as a WL of less than 50% of excess weight, has been described as the most common indication for RMBS [23]. As of January 2024, the IFSO Consensus on Definitions and Clinical Practice Guidelines for Obesity Management established the following reporting standards for specific weight-related definitions, and this includes the adoption of “recurrent weight gain,” signifying a recurrent weight gain > 30% or worsening of an obesity complication that was a significant indication for surgery and “suboptimal initial clinical response” as initial total body weight loss < 20% or inadequate improvement in an obesity complication that was a significant indication for surgery [24].

Other indications for RMBS include persistent reflux or dysphagia, abdominal pain, nausea vomiting, malnutrition, and anatomical complications such as strictures, refractory marginal ulcers, device and hardware complications, fistulas, and internal hernias, among others [25]. Although the initial approach for these complications is usually medical or endoscopic, RMBS may be indicated when initial measures fail. 

### 2.1. Index Procedures

Adjustable gastric banding (AGB) has the highest failure rate of all bariatric procedures, with 20–60% of the patients requiring reoperation [23,26]. IWL is the main indication, accounting for 13.7–62.5% of all the cases [23,25]. Hardware problems, including band slippage, erosion, intolerance, obstruction, and port and tubing problems, are the second most common indication for revision. Band slippage may occur in 1–22% of the patients and accounts for 2–69% of the reoperations [27]. Other indications for revisional surgery include motility problems, infection, and leakage [23,25]. 

Sleeve gastrectomy (SG) is the second procedure that requires the most revisions, with a revision rate that ranges between 14–37% [26]. Again, the best indication is IWL, with an incidence of 13.1% [26]. Similarly, weight recidivism after SG has been reported to be around 27.8% [28]. Other reoperation indications include persistent reflux, and anatomical complications such as anastomotic leaks, sleeve strictures, and sleeve dilation [23].

A minority of RYGBs may require revision. Around 20–24% experience weight regain (WR) after RYGB and may therefore seek a reoperation [26,29,30,31]. Anatomical complications are also a recognized reason for reoperation in these patients; around 1–6% develop gastro-jejunal (GJ) strictures, 0.6–25% marginal ulcers, and 6% gastrogastric fistula after RYGB [32,33]. Although most of these complications can be managed endoscopically, up to 38.5% of GJ strictures and 33.3% of marginal ulcers will require revisional surgery [34,35].

Biliopancreatic diversion (BPD) with or without duodenal switch (DS) and single-anastomosis duodenal–ileal bypass with sleeve gastrectomy (SADI-S) account for around 2% of all the MBS performed in the US [16]. Revision rates are not yet well established, but most revisions are secondary to malnutrition with issues, including persistent diarrhea, metabolic abnormalities, abdominal pain, and persistent emesis [16]. It is expected that revisions for these procedures will increase in the next few years, as more of these surgeries are performed.

### 2.2. Approach to RMBS: Preoperative Evaluation

Patients undergoing RMBS should undergo a multidisciplinary evaluation. At our institution, all patients undergo a comprehensive evaluation by the metabolic and bariatric surgery team, including behavioral and psychosocial assessments and nutritional evaluation. It is important to optimize the patient before the surgery, so micronutrient abnormalities should be corrected before undergoing RMBS [36].

The preoperative workup should also include a contrast-enhanced upper gastrointestinal series (UGI) and upper gastrointestinal (GI) endoscopy to better characterize the anatomy and detect any possible abnormality. Both studies are usually performed in patients undergoing RMBS, as evidenced by a survey answered by 460 bariatric surgeons, where 90% reported performing an upper GI endoscopy and 85.6% a contrasted UGI before surgery [37].

Lastly, depending on the patient’s concomitant symptoms, it may be necessary to request additional studies such as a 24 h pH study in patients with reflux and manometry in patients with dysphagia. Additional images, such as computed tomography (CT) with three-dimensional reconstruction, may also be helpful [38]. 

## 3. Surgical Choice

The choice of revisional procedure mainly depends on the index procedure performed, anatomical complications, and patients’ main complaints. Minimally invasive techniques are preferred over open surgery due to decreased morbidity and mortality [39].

### 3.1. Revisional Gastric Banding

No consensus exists on which procedure should be performed in patients with a failed AGB. Some patients may desire to undergo band removal without any revisional procedure. Endoscopic procedures may also be useful for specific complications such as band erosion [40]. Surgical interventions may achieve more durable results and better weight-related outcomes. The procedure of choice should be decided in consultation with the patient after explaining all the options. This chapter will only discuss the surgical alternatives for revision after AGB. 

Whichever is the revisional surgery of choice, the most important step is band removal. The gastric band produces a fibrotic ring around the stomach, and the careful excision of this ring is a critical step in revisional surgery. Incomplete excision may lead to further complications [41]. There has been discussion over whether revisional surgery for AGB should be performed in one or two steps. Some have reported better outcomes, such as less leakage incidence, with one-stage procedures [42,43]. Therefore, we prefer to perform one-stage operations whenever possible to reduce the number of surgeries and overall cost.

Most surgeons prefer converting to RYGB [37], arguing that as these patients already failed a restrictive procedure, conversion to a procedure with a malabsorptive component makes more sense [44,45]. Some have reported that the WL associated with RYGB conversion is higher and more maintained in time than the one achieved with SG (57.8% EWL for RYGB vs. 22% EWL for SG at 12–24 months) [46]. However, meta-analyses have failed to demonstrate any outcome difference between either procedure [44,45].

Even though WL outcomes may be similar, we prefer RYGB over SG as a standard approach for these patients at our institution. RYGB allows for a better pouch construction, especially in patients with a big anterior pouch [41]. It also permits repair of the scarring and stomach ischemia caused by the band and decreases the risk of gastroesophageal reflux disease (GERD) and migration of the stomach compared to SG [45]. 

The second most used revisional procedure is SG [22], which has gained popularity in the last few years as it may avoid RYGB complications such as dumping syndrome, malabsorption, marginal ulceration, and internal hernia [44]. As previously mentioned, WL outcomes between RYGB and SG and postoperative complications are similar. However, even primary SG has higher reoperation rates than RYGB, mainly due to IWL. Similarly, RYGB has been associated with Barrett’s esophagus regression, so SG should not be the procedure of choice for patients with severe reflux or esophagitis [47]. 

Nevertheless, band conversion to SG is still feasible in patients with preserved anatomy and has good results regarding % EWL and low morbidity. One-anastomosis gastric bypass (OAGB) has become an option for failed AGB [21]. As the OAGB has only one anastomosis, less-severe complication rates have been reported compared to RYGB [48,49]. A recently published matched nationwide study suggested that OAGB as a revisional procedure after AGB has similar short-term WL outcomes as RYGB but better long-term weight results with no difference in postoperative complications [48].

Conversion to DS or SADI-S is also feasible. At our facility, we prefer to perform this surgery in cases where patients have a BMI exceeding 50 kg/m^2^, given the elevated risk of complications following these procedures. Moreover, research indicates that DS yields superior WL outcomes compared to RYGB for patients with a BMI over 50 [50]. Although the results of DS after a failed AGB seem promising, more studies on the topic are needed [46]. 

### 3.2. Revisional Sleeve Gastrectomy 

SG is the most performed MBS nowadays, so it is not surprising that it is the second procedure requiring the most revisions [16]. When patients seek revision for a specific complaint such as persistent reflux or have an anatomical complication like stricture or stenosis, less-invasive treatment options should be considered first. For patients presenting mainly with reflux symptoms, we first prescribe medical therapy with proton pump inhibitors (PPls). If this fails and the patient has symptoms that correlate with 24 h pH monitoring of PPls, we consider them surgical candidates. 

As per a recent Delphi consensus study conducted in 2023, there was a consensus among experts to refrain from employing surgical procedures such as OAGB, banded RYGB, SADI-S, DS, and cruroplasty/hiatal hernia repair as a stand-alone procedure. Additionally, they favored RYGB for managing reflux symptoms post-SG [51].

Similarly, for patients presenting with strictures or stenosis, we first try endoscopic dilation. Again, if the endoscopic treatments fail, they may undergo revisional surgery. 

It is essential for the multidisciplinary team to conduct a comprehensive evaluation of all revision surgeries for GERD, IWL, and WR post-SG. Moreover, patients should receive at least one year of medical and supportive care before undergoing any revision surgeries [51]. Additionally, it is important to emphasize that before proceeding with any revision or conversion surgery, patients are required to undergo a UGI, esophagogastroduodenoscopy (EGD), and functional tests like a manometry as part of our preoperative assessment protocol. 

Surgical alternatives for SG revision include placing a magnetic sphincter augmentation device (MSAD), re-sleeve gastrectomy, RYGB, OAGB, and DS [52,53]. RYGB is most surgeons’ revisional procedure of choice for failed SG [21]. Furthermore, findings from the aforementioned Delphi Consensus study revealed that over 97% of experts favored this procedure [51].

It has been demonstrated that RYGB conversion is feasible, safe, and effective. A meta-analysis reported 68% and 44% EWL 1 and 2 years after RYGB conversion for failed SG [54]. Other studies have reported lower EWL% but still-significant WL outcomes after RYGB conversion with low complication rates [55,56,57,58,59]. 

It has also been demonstrated that RYGB conversion may improve obesity-related comorbidities such as hypertension, diabetes, and obstructive sleep apnea [56,60,61]. Similarly, reflux improves significantly after SG conversion to RYGB [55,56,60,61]. According to a recent study, in a group of patients that underwent RYGB conversion after a failed SG, mean gastroesophageal reflux (GERD) heartburn-related quality of life score (HRQL) significantly decreased in 73% of the patients after 6 months [55]. 

In conclusion, RYGB is used to treat GERD, IWL, and anatomic complications following SG. If the patient’s BMI exceeded 50 kg/m^2^ at the time of RMBS, DS was determined to be the procedure of choice rather than RYGB. IWL was defined as an excess body weight loss of less than 50% at 12 months after primary surgery. GERD was considered an indication for surgery when refractory to 12 to 24 months of clinical therapy and when there was a proven correlation between symptoms and abnormal esophageal acid exposure during 24 h pH monitoring while off PPIs [51]. Anatomic complications after SG included angulations, strictures, and migration of the gastric sleeve into the mediastinum. Endoscopic dilation will be attempted for patients presenting with gastric outlet obstruction.

### 3.3. Revisional Roux-en-Y Gastric Bypass

This procedure is typically recommended for individuals with a BMI of >40 kg/m^2^, or for those patients with a BMI of 35 or higher who also have obesity-related health comorbidities [62]. According to the IFSO Worldwide Survey 2020–2021 on Current Trends for Bariatric and Metabolic Procedures, RYGB is reported as the second most common procedure performed worldwide, following SG [63].

RYGB has been performed with low mortality rates and has demonstrated high efficiency, especially if the impact on WL and comorbidity control is taken into account [64]. Patients usually experience significant WL in a short period of time [65,66]. Compared to SG, RYGB confers superior clinical efficacy in terms of WL and the remission of comorbidities, particularly T2DM [65,66,67]. 

However, with the significant increase in the demand for RYGB for the treatment of obesity, there has also been a rise in post-operative complications following this procedure [68]. In addition, the risk of surgical complications has been demonstrated to be greater following RYGB in comparison to SG [65]. These complications can be further categorized into two groups: early and late complications. Early complications occur within the first 30 days after the surgery and they may include infection, bleeding (1.9–4.4%), intestinal obstruction, suture leaks (0.4–5.2%), deep vein thrombosis (DVT), and pulmonary embolism (PE) [68].

Late complications occur months to years after surgery and they include gastrogastric fistulas (1.5–6%), marginal ulcers (1–16%), strictures (2.9% to 23.0%), nutritional deficiencies, dumping syndrome, and bowel obstruction (adhesions and internal hernia) [68,69].

Nutritional deficiencies, as well as anemia, represent significant long-term complications following RYGB [69,70,71,72]. The altered gastrointestinal anatomy and reduced absorptive capacity resulting from this procedure often lead to inadequate intake and absorption of essential macro and micro-nutrients, including crucial vitamins and minerals such as iron, vitamin B12, calcium, and vitamin D [69,71,72,73]. These deficiencies impact various systems, including the gastrointestinal, cardiac, and neurological systems, among others. They present with a range of symptoms, including fatigue, weakness, dyspnea on exertion, poor wound healing, nausea, constipation, and neurological impairments, significantly impacting patients’ overall well-being and quality of life [71].

Anemia, characterized by diminished red blood cell count or hemoglobin, stands as a prevalent complication that exacerbates symptoms, particularly fatigue and weakness [74]. This condition is directly linked to deficiencies in essential vitamins and minerals, including vitamin B12, folate, and iron, which are frequently observed after RYGB [75,76,77]. Additionally, RYGB patients may encounter protein malnutrition, fat-soluble vitamins (A, D, E, K) deficiencies, alterations in gut microbiota that can lead to small intestinal bacterial overgrowth (SIBO) due to bowel stasis and deficiencies in water-soluble vitamins (B-complex vitamins) and minerals (calcium and iron), further complicating their nutritional status [73,78,79,80].

Management of these multifaceted nutritional deficiencies typically entails lifelong supplementation and dietary modifications. However, despite conservative measures, these complications may persist or worsen over time. In such scenarios, the consideration of revisional, reversal, or conversion procedures as potential interventions to address the underlying etiology may be warranted. However, it is important to note that the existing evidence supporting these interventions is notably limited. Therefore, further rigorous research is imperative to establish guidelines and protocols in this domain [81,82,83].

Lastly, failure of the index procedure includes IWL, WR, and/or re-occurrence of comorbidities.

#### 3.3.1. Management of Late Complications

A commonly recognized complication following RYGB is anastomotic ulcers [84]. They may manifest independently or concurrently with strictures [85]. The primary therapeutic approach involves initiating medical therapy, particularly with sucralfate and PPIs. If ulcers are recurrent or non-healing and cause symptoms that are not relieved by PPIs, this would be regarded as an indication for RMBS, with redo gastrojejunostomy (GJ) being the recommended course of action [85].

When addressing anastomotic strictures, the first course of action typically involves endoscopic dilatation or stent placement, as detailed in the existing literature [86,87]. In cases where endoscopic therapy proves ineffective, revisional surgery, specifically a repeat GJ, may be warranted [85].

A relatively uncommon adverse event is a gastrogastric fistula (GGF). In cases where the GGF is located laterally to the gastric pouch, the pouch is separated from the gastric remnant, and the fistula is divided and closed using a stapler. If the fistula results from a perforated ulcer at the gastro-jejunal anastomosis (GJA) and the perforation extends towards the gastric remnant, it is imperative to redo the GJ, ensuring the separation of the fistula from the remnant. In exceptional cases, a gastrectomy of the remnant may be required [85,88].

#### 3.3.2. Management of WR

WR following RYGB is a critical concern as it can contribute to the recurrence of comorbidities and adversely impact the overall well-being and quality of life of individuals who have undergone the procedure [89]. This adverse event not only poses challenges for long-term weight management but also necessitates comprehensive strategies for monitoring and intervention to mitigate its effects on health outcomes. WR has been associated with several factors, including behavioral, psychological, and anatomical factors, such as the dilation of the GJA which leads to a faster gastric emptying and a reduced sense of satiety [90]. 

From an anatomical standpoint, a dilated or enlarged GJA of >30 mm and the presence of gastrogastric fistula (GGF) serve as noteworthy indicators predictive of weight regain post-RYGB [91,92]. Additionally, from a surgical perspective, a patient may experience IWL as a consequence of pouch stretching, dilation of the stoma, inadequate malabsorption, or other anatomical or physiological issues [93]. 

In our institution, dietary modifications, behavioral interventions, and medications represent the first therapeutic step to treating WR or IWL after a failed RYGB. Patients who met the eligibility criteria for metabolic and MBS based on their body mass index (BMI) and comorbidities (BMI ≥ 40 kg/m^2^ alone or ≥35 kg/m^2^ with metabolic disorders) within 24 months following their initial procedure were assessed for potential revision surgery [94]. 

Re-interventions can pose technical challenges attributed to adhesions and altered anatomy. Furthermore, these reoperations entail a higher risk of complications, morbidity, and mortality in comparison to the primary procedure [90]. According to a newly published report detailing our institution’s experience, late adverse events such as anastomotic stricture were the most common causes of surgical revision, in patients who underwent RYGB as their index procedure, rather than IWL [85]. Nevertheless, when addressing IWL post-RYGB, redo-GJ and pouch resizing procedures are reliable options, albeit with associated risks of significant leak rates [95,96,97]. 

##### Combined TORe and Distalization of the BPL 

Transoral outlet reduction endoscopy (TORe) is a minimally invasive revisional procedure that is proposed as a treatment of WR refractory to conservative therapies [93]. As an endoscopic alternative for more invasive revisional surgeries, TORe primarily focuses on decreasing the size of the GJA in order to delay gastric pouch emptying, thus enhancing the sense of satiety [91,98,99]. Regarding a more technical aspect, TORe involves endoscopic placement of sutures around the dilated GJA, which are then tightened to reduce the anastomotic aperture [100]. 

Several TORe techniques have been developed to improve efficacy, with argon plasma mucosal coagulation (APMC-TORe) and full-thickness suturing plus argon plasma mucosal coagulation (ft-TORe) being the most commonly utilized techniques [98]. As per a meta-analysis conducted by Jaruvongvanich et al., weight reduction outcomes and safety profiles demonstrate comparability between APMC-TORe and ft-TORe procedures [98]. Nevertheless, it is noted that the APMC-TORe procedure typically necessitates multiple endoscopic sessions [98]. 

After undergoing TORe treatment, the total body weight loss (TBWL) was 8.5% in one year (out of 331 patients, *n* = 276), 6.9% in three years (out of 331 patients, *n* = 211), and 8.8% in five years [91]. Furthermore, this study also revealed that most of the patients (77%) stopped gaining weight completely, and 62% of them were still able to maintain a TBWL of greater than 5% after five years [67].

Despite the scarcity of data on this innovative procedure, TORe has displayed a superior safety profile in contrast to traditional surgical revision approaches [67,91]. Moreover, several studies have demonstrated that endoscopic revision of the GJA provides a durable solution for weight management [91,99]. TORe is favored over RMBS for its numerous benefits, including reversibility, faster recovery, and shorter hospital stays [101]. 

Research has shown TORe to be both safe and effective in promoting WL in patients with WR [99,101]. Consequently, TORe, in combination with nutritional, psychological, medical, and endoscopic interventions, emerges as a leading option for managing WR after RYGB, while revisional surgery should be reserved for patients refractory to conservative and minimally invasive treatments [93].

##### Distalization of the BPL

Distalization of the biliopancreatic limb (DBPL) emerges as a strategic intervention targeting both weight-related and metabolic concerns. Distalization aims to optimize the balance between restriction and malabsorption, addressing anatomical and physiological factors that may contribute to the suboptimal outcomes of the primary RYGB. 

In this revisional approach, the biliopancreatic limb is lengthened, and the common channel is shortened to enhance malabsorption and augment WL [102]. Recent studies have found that with a total alimentary limb length (TALL) of 400–450 cm, there was a lower incidence of malnutrition after the procedure [102].

However, as with any surgical intervention, there are associated risks and considerations. Prospective benefits need to be weighed against potential complications, including malnutrition, vitamin deficiencies, and alterations in gastrointestinal function. As such, the decision to pursue DBPL must be individualized, considering the patient’s specific clinical profile, nutritional needs, and the underlying causes of the initial RYGB failure. Close postoperative monitoring and nutritional supplementation are crucial elements to mitigate potential drawbacks and optimize outcomes for patients undergoing this revisional procedure.

##### Conversion of RYGB to DS

Ultimately, two of the most demanding alternatives for revisional surgery are the conversion of RYGB to a biliopancreatic diversion with duodenal switch (BPD/DS), also known as DS, and the conversion to a single-anastomosis duodeno–ileal bypass with sleeve gastrectomy or SADI-S.

Converting to a DS is often recommended for patients presenting with significant comorbidities such as T2DM, hypertension, or metabolic syndrome [103]. This comprehensive approach aims to address multiple health concerns simultaneously, leveraging the DS’s capacity for substantial WL and metabolic improvement [103,104,105]. Conversely, the alternative strategy involves converting to SADI-S. This procedure is a simpler alternative to DS as it involves creating a single anastomosis, potentially decreasing surgical complexity and associated risks of complications.

It is important to outline that the decision whether to convert to a DS or SADI-S is contingent upon the surgeon’s preference and patient-specific considerations. This decision is meticulously tailored to each patient’s unique needs and medical circumstances, while also factoring in the surgeon’s preference, expertise, and judgment. It is a collaborative effort between the patient and the surgical team, ensuring alignment with the patient’s goals, medical history, and desired outcomes. 

Whether the decision is to convert to a DS or SADI-S, both procedures can be conducted either as a one-stage or two-stage process. First, the GJ is disconnected, and a gastro-gastrostomy is constructed to restore gastric continuity. This preparatory phase lays the foundation for the subsequent intervention aimed at optimizing WL outcomes and metabolic control [52].

Subsequently, a DS or SADI-S is performed. This second part of the procedure typically takes place three months after the first stage. Notably, certain authors have noted that performing the procedure in the same setting may result in higher rates of complications or even more severe complications when compared to a two-stage approach [105,106,107]. 

Tran et al. reported that the percentage of EWL after converting RYGB to DS was 62.7% at one year and 71% at three years, showing significant and sustained WL [100]. According to a related study, the DS showed the highest amount of WL after a 5-year follow-up when compared to other bariatric procedures [89]. Other studies have shown that many patients experience improvement or complete resolution of conditions such as T2DM, high blood pressure, and sleep apnea, with a reduced risk of WR [108,109,110].

However, there are potential drawbacks and considerations associated with this procedure. One notable concern is the increased risk of malabsorption-related complications, including nutrient deficiencies and metabolic imbalances necessitating vigilant monitoring and lifelong nutritional supplementation [111]. Additionally, the potential for gastrointestinal symptoms, such as diarrhea or steatorrhea, poses a challenge for some patients undergoing this conversion, impacting their quality of life [111].

### 3.4. Revisional Duodenal Switch (DS)

DS is one of the most challenging interventions in terms of technique, recovery time, and chances of malnutrition [112]. It combines restrictive and malabsorptive techniques and is typically recommended for individuals with a BMI over 50 kg/m^2^. 

This procedure consists of an SG followed by a duodeno-ileostomy and an ileo-ileostomy, creating a long Roux-en-Y with a 150 cm alimentary limb and a 120 cm common channel [113]. Comparably, SADI-S is a variation of the DS procedure, which involves the creation of a single anastomosis connecting the post-pyloric portion of the duodenum to the small intestine, 250 cm proximal to the ileocecal valve [114]. 

Despite its efficacy in promoting substantial WL and addressing obesity-related comorbidities, this procedure presents considerable challenges [115,116]. Numerous studies have described malabsorption as the predominant complication following SADI-S and DS interventions, resulting in consequential malnutrition, with prevalence rates ranging from 2% to 18.5% [117,118,119]. However, recent studies have identified GERD as a post-procedural side effect following SADI-S and DS [120,121,122,123,124].

Additionally, given the complex nature of the surgery, DS may lead to a higher risk of perioperative complications, requiring longer hospital stay when compared to other MBS [111,115,116,125]. Moreover, a high degree of skill is required to perform this procedure, and patients frequently have prolonged recovery times that need meticulous postoperative care [115,125]. 

#### 3.4.1. Management of Gastroesophageal Reflux Disease

In the assessment of patients who have developed GERD after SADI-S or DS procedures, a comprehensive diagnostic approach is undertaken at our institution. Initial imaging studies, including EGD, UGI, and esophageal manometry examinations, are performed to discern any underlying anatomical anomalies, such as hiatal hernia, sleeve narrowing, or esophageal dysmotility, that may contribute to the patient’s symptoms.

Subsequently, functional assessments, such as the Bravo pH study and 24 h pH-impedance test, are conducted to evaluate the extent of acid and bile exposure and quantify reflux events, respectively [126]. These diagnostic tools provide crucial insights into the pathophysiology of GERD in each patient, guiding subsequent therapeutic decisions.

**Figure 1:**
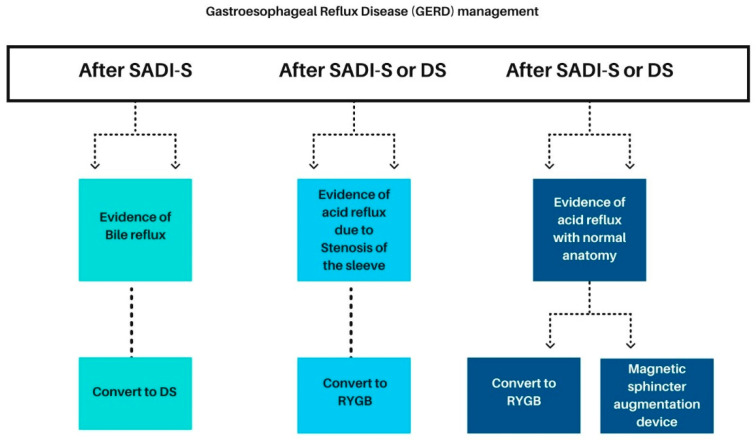
Gastroesophageal reflux disease (GERD) management after SADI-S or DS. SADI-S single-anastomosis duodeno–ileal bypass with sleeve gastrectomy. DS, duodenal switch; RYGB, Roux-en-Y gastric bypass.

For patients presenting with GERD symptoms despite normal anatomical findings in preoperative assessments, conversion to RYGB or placing an MSAD are viable management options [127,128] (Figure 1). It is important to mention that before the implantation of the MSAD, preoperative esophageal manometry is recommended to assess for any underlying esophageal pathology.

In cases where bile reflux is identified in a 24 h pH-impedance study following SADI-S, conversion to conventional DS may be warranted [129,130].

Similarly, patients who develop GERD due to sleeve stenosis post-SADI-S or DS may benefit from conversion to RYGB to address the underlying issue [131,132,133].

If a hiatal hernia is identified as the underlying cause of the patient’s reflux symptoms, it should be promptly addressed. Simultaneous repair of any pathological hiatal hernia during the aforementioned procedures is recommended to optimize outcomes and minimize the risk of symptom recurrence.

This comprehensive approach ensures tailored management strategies for patients with post-MBS GERD, addressing both anatomical abnormalities and functional disturbances to achieve optimal clinical outcomes.

#### 3.4.2. Management of Malabsorption

Malnutrition after MBS refers to a condition where individuals experience deficiencies in essential nutrients, vitamins, and minerals following MBS. This can occur due to numerous factors such as reduced food intake, alterations in nutrient absorption, changes in dietary habits, and inadequate supplementation [134].

Malnutrition can manifest in different forms, including deficiencies in protein, vitamins such as B12, D, and folate, minerals such as iron, calcium, and zinc, and macronutrients such as carbohydrates, fats, and proteins [134]. If left untreated, malnutrition can lead to serious health complications, including weakness, fatigue, impaired wound healing, osteoporosis, neurological and gastrointestinal problems, and cardiovascular, renal, and immune system dysfunction [134].

To address malabsorption-related complications, some variations of the DS incorporate a side-to-side anastomosis, often referred to as “kiss anastomosis” [135]. This involves connecting the biliopancreatic limb (BL) and the common channel (CC) in a side-to-side fashion, forming a loop while lengthening both limbs and allowing pancreatic enzymes and bile to mix with the food further down the small intestine [135]. By incorporating this anatomical modification, the side-to-side anastomosis is designed to decrease the likelihood of bile reflux into the esophagus, thereby reducing the occurrence of malabsorption-related complications. Furthermore, this altered anatomical configuration can improve the absorption of vital nutrients, alleviating concerns associated with malabsorption. Essentially, the side-to-side anastomosis aims to achieve a balance between significant WL and the mitigation of complications [135].

Another strategy to manage malnutrition is by suppressing the malabsorptive part of the biliopancreatic diversion with a proximal anastomosis [117]. This procedure generally refers to a connection between the biliopancreatic limb and the alimentary limb made at a higher anatomical point than the traditional DS anastomosis, resulting in the creation of a “dual channel” and the restoration of a completely functional small bowel [117]. Despite these modifications aiming to create a more balanced physiological environment, the potential for WR and early and late morbidity remains [118].

In summary, these techniques share a common goal of refining the DS procedure to enhance its efficacy and minimize complications, particularly those related to reflux and malabsorption. It is crucial to acknowledge, however, that the specific nuances of each technique, including the anatomical location of the anastomosis and the degree of limb rearrangement, can vary among surgeons and institutions. When using these procedures, surgeons should place a high priority on having a complete understanding of the anatomical subtleties and possible effects of each alteration. Individualized patient assessment, including meticulous consideration of pre-existing conditions and long-term nutritional implications, remains paramount.

## 4. Conclusions

RMBS presents a complex landscape fraught with challenges and considerations. The primary hurdles involve addressing the complications arising from the initial bariatric procedure. Striking a delicate balance between achieving sustainable WL and minimizing potential risks remains a formidable challenge. Patient selection becomes pivotal, as individualized approaches are necessary to tailor interventions to specific clinical profiles and needs while maximizing patient outcomes. Furthermore, the involvement of a multidisciplinary team of experts is imperative in navigating this intricate terrain. Their collaboration ensures thorough pre-operative evaluation, precise surgical intervention, and attentive post-operative care. Additionally, given the complexity of these cases, RMBS should ideally be performed at specialized centers equipped with the requisite infrastructure and experience to manage such procedures effectively. Adhering to these principles facilitates the optimization of outcomes while minimizing risks for patients undergoing RMBS.

## Data Availability

The original data presented in the study are openly available in PubMed at [https://pubmed.ncbi.nlm.nih.gov/].
